# Albumin-bound paclitaxel and gemcitabine combination therapy in soft tissue sarcoma

**DOI:** 10.1186/s12885-020-07199-0

**Published:** 2020-07-28

**Authors:** Zhichao Tian, Fan Zhang, Po Li, Jiaqiang Wang, Jinpo Yang, Peng Zhang, Weitao Yao, Xin Wang

**Affiliations:** 1 Department of Bone and Soft Tissue, the Affiliated Cancer Hospital of Zhengzhou University and Henan Cancer Hospital, Zhengzhou, 450008 Henan Province China; 2 Department of Medical Oncology, the Affiliated Cancer Hospital of Zhengzhou University and Henan Cancer Hospital, Zhengzhou, 450008 Henan Province China

**Keywords:** Albumin-bound paclitaxel, Gemcitabine, Sarcoma, Epithelioid sarcoma, Chemotherapy

## Abstract

**Background:**

The evidence that albumin-bound paclitaxel (nab-paclitaxel) is safe and efficacious for the treatment of many types of malignant tumors is continuously increasing. However, the evidence and clinical data of nab-paclitaxel and gemcitabine in metastatic soft tissue sarcoma (STS) treatment are rare.

**Methods:**

The clinical data of metastatic STS patients who received nab-paclitaxel/ gemcitabine chemotherapy between January 2019 and February 2020 were retrospectively analysed. All these patients were treated with nab-paclitaxel/ gemcitabine only after doxorubicin-based chemotherapy had failed. We evaluated the effectiveness and safety of nab-paclitaxel and gemcitabine in these patients.

**Results:**

A total of 17 patients treated with nab-paclitaxel/ gemcitabine were enrolled in this study. One patient with angiosarcoma achieved complete response, 6 patients had partial response, 5 patients achieved stable disease, and 5 patients had progressive disease. The average diameter change in target lesion from baseline was − 19.06 ± 45.74%. And median progression free survival was 6 months (95% CI, 2–9 months). Grade 3 / 4 adverse events were not common, included neutropenia (17.6%), fatigue (11.8%), anemia (11.8%), leukopenia (11.8%), nausea (5.9%), peripheral neuropathy (5.9%), diarrhea (5.9%), and thrombocytopenia (5.9%). No treatment-related deaths occurred.

**Conclusion:**

Nab-paclitaxel/ gemcitabine combination chemotherapy is comparatively effective in the treatment of STS, demonstrates low toxicity, and is worthy of further study.

## Background

Soft tissue sarcomas (STS) comprise a diverse family of malignancies predominantly of mesodermal origin. Although the incidence of STS is low, there are still more than 40,000 new cases in China each year [1]. After standard treatment is completed, most cases of STS eventually progress into locally unresectable or metastatic advanced STS. The first-line treatment for unresectable or metastatic STS is doxorubicin-based chemotherapy, with an expected overall response rate (ORR) of 11–26% and a median progression free survival (median-PFS) of 4–8 months [2, 3]. Docetaxel/ gemcitabine is another common chemotherapy regimen, often considered a second line treatment (after doxorubicin), with an expected ORR and an median-PFS similar to those of doxorubicin [4, 5]. Multi-target receptor tyrosine kinase inhibitors (TKIs) and programmed cell death protein 1 (PD-1) inhibitors have also been shown to be effective against selective STS, although with a lower ORR and median-PFS than those of doxorubicin and docetaxel/ gemcitabine [6, 7]. Because of the limited ORR and median-PFS obtained with each of the above treatments, the overall survival for advanced STS is approximately only 16 months [8]. Therefore, there is an urgent need for more effective drugs to treat STS.

Albumin bound paclitaxel (nab-paclitaxel) is a new ethanol-free paclitaxel, and was initially developed to overcome toxicities associated with the solvents used in conventional formulations and to potentially improve efficacy [9]. Nab-paclitaxel has been shown to deliver a higher dose of paclitaxel to tumor lesions (compared to solvent-based paclitaxel formulations) and to decrease the incidence of serious toxicities, including severe allergic reactions [10, 11]. Up to now, nab-paclitaxel has been approved for the treatment of metastatic pancreatic cancer, locally advanced or metastatic non-small cell lung cancer, and metastatic breast cancer [10, 12]. Not only that, there is growing evidence that nab-paclitaxel is effective in the treatment of other malignant tumors [13, 14], including STS [15–17].

Since January 2019, advanced sarcoma patients have been treated in our hospital (a major sarcoma treatment center in central China) with nab-paclitaxel and gemcitabine. In this study, we retrospectively investigate patient outcomes and study the safety and effectiveness of nab-paclitaxel/ gemcitabine combination chemotherapy in STS treatment, with the aim of providing additional evidence to establish clinical study design and to support clinical treatment.

## Methods

### Patients and eligibility criteria

All the STS patients in this retrospective study received nab-paclitaxel/ gemcitabine combination chemotherapy between January 2019 and February 2020. This study was approved by the Ethics Committee of The Affiliated Cancer Hospital of Zhengzhou University. All patients provided written informed consent for data collection and research purposes. The inclusion criteria were as follows: 1) histologically proven STS; 2) locally unresectable or multiple metastases; 3) treated with nab-paclitaxel/ gemcitabine chemotherapy; 4) measurable lesions according to the Response Evaluation Criteria in Solid Tumors (RECIST, version 1.1); 5) The clinical data are complete and can be statistically analyzed.

### Treatment protocol

Patients were administered 300 mg/m^2^ nab-paclitaxel via intravenous bolus on day 1, and 1250 mg/m^2^ gemcitabine via intravenous bolus on days 1 and 8. All patients received a single sub-cutaneous injection of polyethylene glycol recombinant human granulocyte colony- stimulating factor 100 μg/kg on day 3. The treatment regimen was repeated every 21 days, until manifestation of progressive disease (PD) or unacceptable adverse events (AEs). If grade 3 / 4 AEs occurred, treatment was delayed until recovery. However, if the delay lasted more than 14 days, the treatment was terminated.

### Evaluation of effectiveness and safety

The baseline characteristics of all STS patients enrolled in this study were reviewed. Treatment effectiveness was evaluated according to the RECIST (version 1.1) criteria every 1 or 2 months using either magnetic resonance imaging or computed tomography. The ORR, disease control rate (DCR), median-PFS and AEs were then evaluated. ORR and DCR were defined based on RECIST (version 1.1). The National Cancer Institute’s Common Terminology Criteria for Adverse Events (version 4.0) was used to evaluated AEs. PFS was defined as the time from initiation of drug treatment to the date of PD or death, and survivors without PD were censored at the last contact.

### Statistical analyses

All data were analyzed using SPSS 21.0 software. The present study comprises a descriptive analysis. Quantitative variables are presented as numerical values (percentage) and medians (range). The corresponding figure was drawn using GraphPad Prism 5.0. PFS was calculated using the Kaplan-Meier method, with a 95% confidence interval (CI).

## Results

### Patient characteristics

A total of 17 STS patients treated with nab-paclitaxel/ gemcitabine were enrolled in this study. The characteristics of these patients are shown in Table 1. The cohort included 11 (64.71%) men and 6 (35.29%) women. The average patient age was 38.71 ± 17.35 years. All patients had stage IV disease. All patients were previously treated with doxorubicin-based or other chemotherapy. The primary tumor site varied significantly. and although primary tumor sites were distributed throughout the body, they were mainly situated in the extremities. There were also markedly differences in histological subtypes. The most common subtype being epithelioid sarcoma (*n* = 5), followed by angiosarcoma (*n* = 3), rhabdomyosarcoma (*n* = 3), undifferentiated pleomorphic sarcoma (*n* = 2), fibrosarcoma(*n* = 2), leiomyosarcoma (*n* = 1), and primitive neuroectodermal tumor (*n* = 1).

**Table 1:** Patient demographics and characteristics

Patient No.	ECOG PS	Histological subtype	Stage	Primary site	Metastatic site	Previous DOX chemotherapy	Response	PFS (Months)
1	0	Epithelioid sarcoma	IV	Extremities	Lung and bone	Yes	PR	6
2	1	Epithelioid sarcoma	IV	Extremities	Lung and lymph nodes	Yes	PR	11
3	1	Epithelioid sarcoma	IV	Extremities	Lung and bone	Yes	PR	6
4	0	Epithelioid sarcoma	IV	Extremities	Bone	Yes	SD	9
5	1	Epithelioid sarcoma	IV	Extremities	Lung	Yes	PD	1.5
6	1	Angiosarcoma	IV	Extremities	Bone	Yes	CR	12
7	0	Angiosarcoma	IV	Extremities	Lung	Yes	SD	8
8	0	Angiosarcoma	IV	Extremities	Lung	Yes	PR	7
9	0	Rhabdomyosarcoma	IV	Head	Lung and Soft tissue	Yes	PR	9
10	0	Rhabdomyosarcoma	IV	Trunk	Bone	Yes	SD	6
11	0	Rhabdomyosarcoma	IV	Extremities	Lung	Yes	PD	3
12	1	UPS	IV	Extremities	Lung	Yes	SD	6
13	0	UPS	IV	Pelvis	Lung	Yes	PD	1.3
14	1	Fibrosarcoma	IV	Trunk	Lung	Yes	PR	9
15	0	Fibrosarcoma	IV	Extremities	Lung	Yes	PD	1
16	0	Leiomyosarcoma	IV	Extremities	Lung	Yes	PD	2
17	1	PNET	IV	Trunk	Lung	Yes	SD	3

*Abbreviations*: *ECOG PS* Eastern Cooperative Oncology Group performance status, *UPS* Undifferentiated pleomorphic sarcoma, *PNET* Primitive neuroectodermal tumor, *DOX* Doxorubicin-based, *PR* Partial response, *SD* Stable disease, *PD* Progressive disease, *CR* Complete response. *PFS* Progression-free survival

### Effectiveness of therapy

**Fig. 1:**
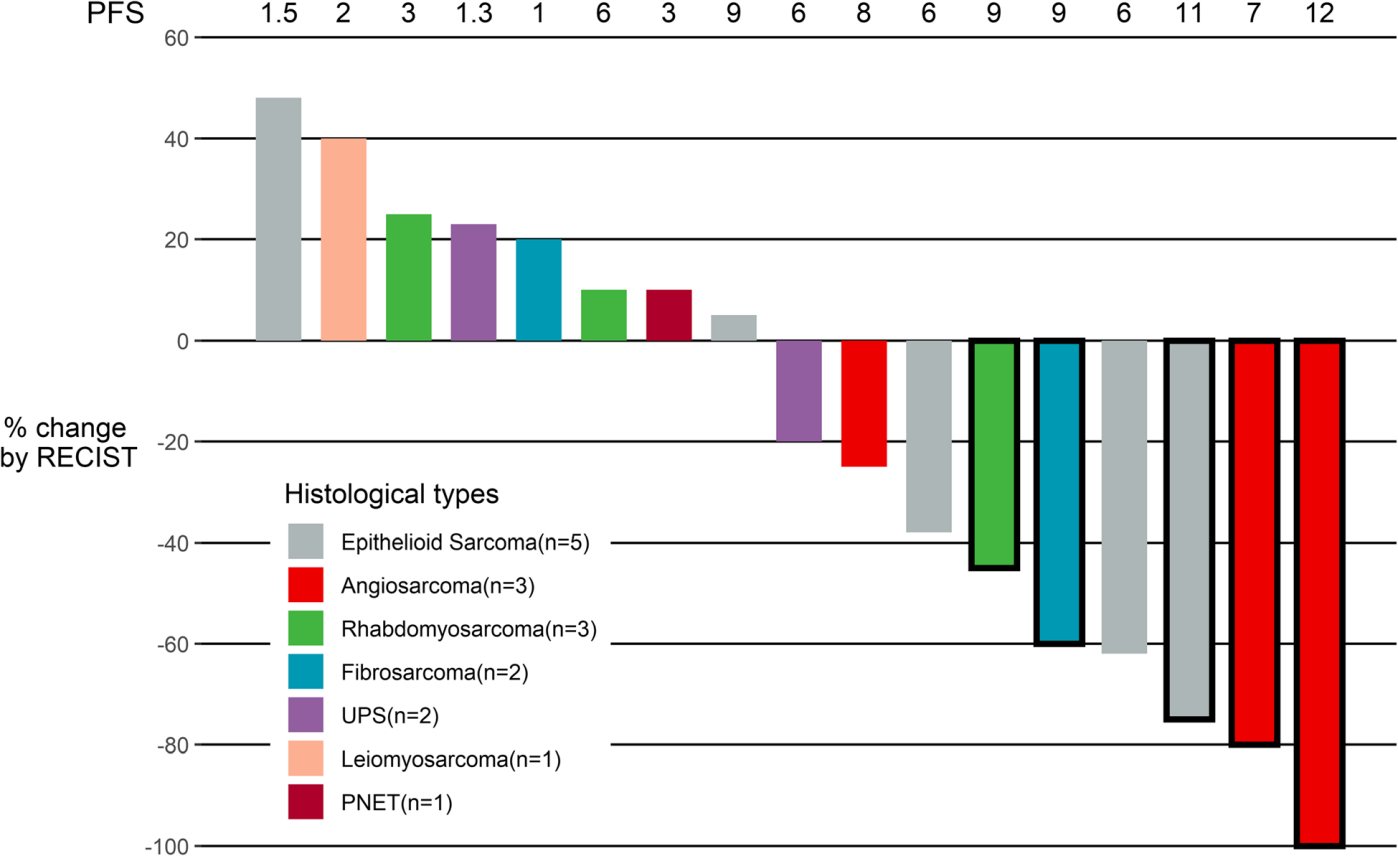
The maximum percentage diameter changes from baseline in target lesion. The effectiveness was evaluated according to the Response Evaluation Criteria in Solid Tumors version 1.1 (RECIST). *PFS* the progression-free survival, *UPS* undifferentiated pleomorphic sarcoma, *PNET* primitive neuroectodermal tumor

Of the 17 patients, 1 patient with angiosarcoma achieved complete response, 6 patients had partial response, 5 patients achieved stable disease (SD), and 5 patients had PD (Fig. 1; Tables 2). The average diameter change from baseline in target lesion was − 19.06 ± 45.74% (Fig. 1). The ORR was 41.2%, the DCR was 70.6%, the median-PFS was 6 months (95% CI, 2–9 months), and the 6-months PFS rate was 64.71% (Table 3; Fig. 1).

**Table 2:** Responses of various histological subtypes to treatment

Histological subtypes	Number of patients			
	CR	PR	SD	PD
Epithelioid sarcoma (*n* = 5)	0	3	1	1
Angiosarcoma (*n* = 3)	1	1	1	0
Rhabdomyosarcoma (*n* = 3)	0	1	1	1
UPS (*n* = 2)	0	0	1	1
Fibrosarcoma (*n* = 2)	0	1	0	1
Leiomyosarcoma	0	0	0	1
PNET	0	0	1	0
Total	1	6	5	5

*Abbreviations*: *CR* Complete response, *PR* Partial response, *SD* Stable disease, *PD* Progressive disease, *UPS* Undifferentiated pleomorphic sarcoma, *PNET* Primitive neuroectodermal tumor

**Table 3:** Clinical effectiveness

Characteristics	Data
ORR	41.20%
DCR	70.60%
M-PFS (months)	6 (95%CI: 2–9)
6 months PFS rate	64.71%

Data are presented as percentages or means

*Abbreviations*: *ORR* The objective response rate, *DCR* The disease control rate, *m-PFS* The median progression-free survival

### Toxicity and safety

In general, nab-paclitaxel/ gemcitabine chemotherapy was relatively well tolerated. As shown in Table 4, the most common grade 1or 2 AEs were alopecia (88.2%, 15/17), neutropenia (64.7%, 11/17), fatigue (52.9%, 9/17), anemia (47.1%, 8/17), and nausea (41.2%, 7/17). The grade 3 or 4 AEs were not common, but included neutropenia (17.6%), fatigue (11.8%), anemia (11.8%), leukopenia (11.8%), nausea (5.9%), peripheral neuropathy (5.9%), diarrhea (5.9%), and thrombocytopenia (5.9%). No treatment-related deaths occurred.

**Table 4:** Adverse events

Adverse events	Grade 1–2	Grade 3–4
Alopecia	88.2% (15/17)	
Neutropenia	64.7% (11/17)	17.6% (3/17)
Fatigue	52.9% (9/17)	11.8% (2/17)
Anemia	47.1% (8/17)	11.8% (2/17)
Nausea	41.2% (7/17)	5.9% (1/17)
Leukopenia	35.3% (6/17)	11.8% (2/17)
Peripheral neuropathy	29.4% (5/17)	5.9% (1/17)
Anorexia	29.4% (5/17)	
Diarrhea	23.5% (4/17)	5.9% (1/17)
Thrombocytopenia	23.5% (4/17)	5.9% (1/17)
Alkaline phosphatase increased	17.6% (3/17)	
Fever	11.8% (2/17)	
Abdominal pain	11.8% (2/17)	
Pneumonitis	5.9% (1/17)	

Data are presented as percentages (number events/total)

## Discussion

The taxanes (including paclitaxel and docetaxel) represent a class of chemotherapy drugs that interfere with microtubule function leading to altered mitosis and cellular apoptosis [18]. Paclitaxel is rarely used in the treatment of STS because it demonstrates limited efficacy [19]. The main reason for the limited efficacy of paclitaxel is probably that there is a practical limitation to the delivered dose due to high toxicity [10, 20–22]. The toxicity of docetaxel-based chemotherapy is greater than that of doxorubicin-based chemotherapy. Thus, although the clinical efficacy of the two regimens is similar, docetaxel-based chemotherapy is considered as a second line regimen for the treatment of advanced STS [4].

Treatment with paclitaxel or docetaxel is associated with a number of clinical problems, including poor drug solubility, allergic reactions, serious dose limiting toxicities [18]. These clinical problems are related to the solvents used to dilute these anticancer drugs: Cremophor EL for paclitaxel and polysorbate 80 for docetaxel [22]. To solve these problems, nab-paclitaxel was developed to be free of the conventional solvents used in the injections. The newly nab-paclitaxel is prepared by encapsulating paclitaxel in albumin nanoparticle [9]. The nab-paclitaxel can pass through the leaky capillary junctions in the tumor bed more easily than through the normal vessels in healthy tissue, and is thus taken up selectively by tumor tissues and cells. According to clinical data, nab-paclitaxel offers several improvements over conventional, solvent- and Cremophor-based paclitaxel, including lower toxicities, shorter administration time, higher efficacy, and the lack of a need for premedication [23]. Several previous studies have demonstrated that nab-paclitaxel has greater efficacy and a more favorable safety profile (compared with solvent-based paclitaxel) in many malignancies [13, 24, 25].

To our knowledge, this study is the first to investigate the safety and effectiveness of nab-paclitaxel/ gemcitabine combination chemotherapy in advanced STS patients. In this retrospective observational study, we observed that nab-paclitaxel was effective for the treatment of STS, with an ORR of 41.2%, and a median-PFS of 6 months. Considering that all of these patients received nab-paclitaxel/ gemcitabine treatment following failed doxorubicin-based chemotherapy, the effectiveness of nab-paclitaxel/ gemcitabine chemotherapy is increased compared to doxorubicin-based chemotherapy and docetaxel/ gemcitabine combination therapy reported by other studies [2, 4]. The toxicity of nab-paclitaxel/ gemcitabine is also lower than that of doxorubicin and docetaxel/ gemcitabine [4]. In addition, the results of this study demonstrate that the effectiveness of nab-paclitaxel/ gemcitabine is significantly greater than that of conventional paclitaxel and docetaxel in some subtypes of STS, such as epithelioid sarcoma. Previous studies have demonstrated the limited efficacy of conventional paclitaxel and docetaxel in the treatment of epithelioid sarcoma [26, 27]. In the present study, three of the five epithelioid sarcoma patients achieved PR, and one patient achieved SD.

The results of our study indicate that nab-paclitaxel is more effective and has lower toxicity than conventional paclitaxel or docetaxel in STS. In view of the fact that nab-paclitaxel is superior to conventional paclitaxel in the treatment of many malignant tumors [13, 24, 25], nab-paclitaxel should not simply be considered as a drug with similar properties to paclitaxel. It should be regarded as a new chemotherapeutic drug; and the efficacy of this drug should be evaluated in various malignancies. For example, though paclitaxel is considered to be ineffective in the treatment of osteosarcoma [28], it should not be assumed that treatment of osteosarcoma with nab-paclitaxel is also ineffective. Indeed, we speculate that treatment of osteosarcoma with nab-paclitaxel may yield promising results.

This study provides preliminarily results demonstrating the safety and effectiveness of nab-paclitaxel/ gemcitabine in STS treatment. Although this study has some limitations, including the small sample size, retrospective design and the lack of a control group, we can still conclude that nab-paclitaxel/ gemcitabine combination chemotherapy used in STS treatment demonstrates promising effectiveness with low toxicity, and is worthy of further study. In view of the low toxicity and convenience of nab-paclitaxel, we believe that the combination of nab-paclitaxel and other anticancer drugs (chemotherapeutic drugs, TKIs, PD-1 inhibitors) in the treatment of STS may produce significant results. In elderly sarcoma patients, where effective treatment is wanting due to poor tolerance [29], nab-paclitaxel may be of significant benefit. Fortunately, several clinical trials on the efficacy of nab-paclitaxel in sarcomas are currently recruiting patients (Table 5). To further investigate the efficacy of nab-paclitaxel in STS, a randomized clinical trial will be conducting soon (ChiCTR2000030250).

**Table 5:** Clinical trials of nab-paclitaxel in sarcomas currently recruiting

Title	Phase	Status	Histological subtypes	Number of patients	Collaborators	Dates	NCT Number
Nab-paclitaxel and gemcitabine in advanced STS.	1 and 2	Recruiting	STS	45	Swiss Group for Clinical Cancer Research	Start: October 2018 Completion: October 2022	NCT03524898
Nab-paclitaxel and gemcitabine for recurrent/refractory sarcoma.	2	Recruiting	Osteosarcoma, Ewing Sarcoma, Rhabdomyosarcoma, STS	72	H. Lee Moffitt Cancer Center and Research Institute National Pediatric Cancer Foundation	Start: October 2016 Completion: March 2021	NCT02945800
Trial of nab-paclitaxel in patients with desmoid tumors and multiply relapsed/refractory desmoplastic small round cell tumors and Ewing sarcoma.	2	Recruiting	Desmoid tumors, Desmoplastic small round cell tumors, Ewing sarcoma	61	Grupo Espanol de Investigacion en Sarcomas	Start: May 2017 Completion: September 2020	NCT03275818
Nab-paclitaxel in combination with gemcitabine for pediatric relapsed and refractory solid tumors.	1	Recruiting	Dediatric relapsed and refractory solid tumors	24	Emory University, Celgene Corporation	Start: August 2018 Completion: May 2022	NCT03507491

*Abbreviations*: *nab-paclitaxel* Albumin-bound paclitaxel, *STS* Soft tissue sarcoma

## Conclusions

In conclusion, nab-paclitaxel/ gemcitabine combination chemotherapy is comparatively effective in STS treatment, demonstrates low toxicity, and is worthy of further study.

## Data Availability

The datasets used and/or analysed during the current study are available from the corresponding author on reasonable request.

## Abbreviations

Nab-paclitaxel: Albumin-bound paclitaxel
STS: Metastatic soft tissue sarcoma
median-PFS: Median progression-free survival
DCR: Disease control rate
ORR: Objective response rate
AEs: Adverse events
TKIs: Receptor tyrosine kinase inhibitors
PD-1: Programmed cell death protein 1
RECIST: Response Evaluation Criteria in Solid Tumors
PD: Progressive disease
SD: Stable disease
ECOG PS: Eastern Cooperative Oncology Group performance status
UPS: Undifferentiated pleomorphic sarcoma
PNET: Primitive neuroectodermal tumor
DOX: Doxorubicin-based
PR: Partial response
CR: Complete response
